# Supplementary material for: Angular velocity integration in a fly heading circuit
Source: eLife. 2017 May 22;6:e23496. doi: 10.7554/eLife.23496 (PMC5440168; doi:10.7554/eLife.23496)
Supplement: Supplementary file 1. — Listed are, for all in vivo electrophysiology experiments, the fly’s genotype, experimental condition, time spent walking, mean forward, absolute sideslip, and absolute rotational velocity as well as the range of rotational velocities displayed by the fly while walking. Spike rate and membrane potential (Vm) at rest were determined for non-walking periods, maximum spike rates are computed for 50 ms windows. All previous parameters are reported as mean and standard deviation across one-minute windows of the entire experiment. The spike threshold is the membrane potential at the peak of the second derivative of the membrane potential before a spike and was determined for a subset of spikes measured at resting membrane potential. The apparent input resistance was computed from a single exponential fit to the decay phase of a depolarizing current pulse. The bias current is a constant current injected to compensate for the leak through the seal resistance, in some experiments this current was readjusted during the recording. DOI: http://dx.doi.org/10.7554/eLife.23496.026 [file elife-23496-supp1.docx]

| **exp** | **genotype** | **condition** | **Walking [%]** | **v_Rot_ [°/s]** | **v_Rot_ range [°/s]** | **vForward**  **[mm/s]** | **vSideslip** | **Spike rate at rest [Hz]** | **Max. spike rate [Hz]** | **Vm at rest [mV]** | **Spike threshold [mV]** | **R_input_ [GΩ]** | **Holding current** |
| --- | --- | --- | --- | --- | --- | --- | --- | --- | --- | --- | --- | --- | --- |
| 1 | 37F06 GFP | visual closed loop | 52±7 | 31±4 | 230±42 | 0.38±0.12 | 0.98±0.1 | 0.2±0.2 | 41.3±13.6 | -43±2.5 | -25±1.9 | 2.2  [2.2, 2.2] | -0.7±0.1 |
| 2 | 37F06 60D05L LCsChr | darkness | 43±18 | 24±6 | 210±58 | 0.31±0.17 | 0.9±0.2 | 3.4±1.5 | 52.5±19.1 | n.a. | n.a. | n.a. | n.a. |
| 3 | 37F06L 55G08 CsChrimson | darkness | 70±4 | 44±2 | 380±19 | 0.66±0.11 | 1.7±0 | 7.5±1.0 | 80±28.3 | n.a. | n.a. | n.a. | n.a. |
| 4 | 37F06 GFP | darkness | 81±8 | 30±0 | 200±29 | 0.54±0.10 | 1.3±0.1 | 1.2±0.9 | 40±20 | n.a. | n.a. | n.a. | n.a. |
| 5 | 37F06 GFP | visual closed loop | 88±10 | 29±4 | 240±90 | 2.5±0.85 | 2.2±0.3 | 1.4±1.6 | 54.7±16.2 | -47±3.2 | -34±5.4 | 2.0  [2, 2.1] | -4.8±0.0 |
| 6 | 37F06 GFP | darkness | 54±10 | 15±1 | 120±29 | 0.44±0.10 | 0.7±0.1 | 3.7±1.6 | 50±10.5 | n.a. | n.a. | n.a. | n.a. |
| 7 | 37F06L LGFP 55G08 Jaws-mCherry | visual closed loop | 43±21 | 11±2 | 130±39 | 0.03±0.1 | 0.6±0.1 | 1.6±0.4 | 31.1±10.5 | -48±1.8 | -38±3.5 | 1.72 [1.7, 1.8] | -8.4±0.5 |
| 8 | 37F06L LGFP 76E11 Jaws-mCherry | darkness | 54±15 | 15±2 | 170±51 | 0.18±0.1 | 0.7±0.1 | 2.6±0.5 | 55.6±8.8 | -42±1.6 | -34±9.2 | 1.7  [1.7, 1.8] | -7.5±0.2 |
| 9 | 37F06L LGFP 55G08 Jaws | visual closed loop | 53±18 | 17±5 | 170±50 | 0.12±0.19 | 0.7±0.2 | 1.7±0.6 | 34±9.7 | -57±0.93 | -41±1.6 | 0.8  [0.7, 0.9] | -10±0.0 |
| 10 | 37F06L LGFP 55G08 Jaws-mCherry | visual closed loop | 47±14 | 18±4 | 190±51 | 0.07±0.07 | 0.6±0.1 | 1.4±0.5 | 42.2±6.7 | -44±1.4 | -30±2 | 1.8  [1.8, 1.9] | -9.9±0.0 |
| 11 | 37F06 LGFP 76E11 Jaws | visual closed loop | 27±14 | 19±6 | 150±53 | 0.28±0.23 | 0.7±0.3 | 2.4±1.0 | 36.4±8.1 | -45±1.1 | -35±1.3 | 1.1  [1.1, 1.1] | -5.9±0.1 |
| 12 | 37F06 GFP | darkness | 42±9 | 19±8 | 180±38 | 0.17±0.05 | 0.5±0.1 | 5.3±2.0 | 53.3±14.1 | -43±3.4 | -36±8.1 | 3.6  [3.3, 3.9] | -3.6±3.1 |

**Table 1 – Recording parameters of in vivo electrophysiology**

Listed are, for all in vivo electrophysiology experiments, the fly’s genotype, experimental condition, time spent walking, mean forward, absolute sideslip, and absolute rotational velocity as well as the range of rotational velocities displayed by the fly while walking. Spike rate and membrane potential (Vm) at rest were determined for non-walking periods, maximum spike rates are computed for 50 ms windows. All previous parameters are reported as mean and standard deviation across one minute windows of the entire experiment. The spike threshold is the mean and standard deviation of the membrane potential at the peak of its second derivative preceding a spike, it was determined for a subset of spikes measured at resting membrane potential. The apparent input resistance was computed from a single exponential fit to the decay phase of a depolarizing current pulse, it is reported as mean and confidence bounds. The bias current is a constant current injected to compensate for the leak through the seal resistance, in some experiments this current was readjusted during the recording, reported is the mean and standard deviation across the whole experiment.
