# Supplementary material for: Angular velocity integration in a fly heading circuit
Source: eLife. 2017 May 22;6:e23496. doi: 10.7554/eLife.23496 (PMC5440168; doi:10.7554/eLife.23496)
Supplement: Supplementary file 2. — Listed are, for all in vivo calcium imaging experiments, the fly’s genotype, temperature (in the case of the shiTS flies), percentage of time spent walking, mean forward, absolute sideslip, and absolute rotational velocity as well as the range of rotational velocities displayed by the fly while walking. DOI: http://dx.doi.org/10.7554/eLife.23496.027 [file elife-23496-supp2.docx]

| **fly** | **genotype** | **Walking [%]** | **vRot [°/s]** | **vForward [mm/s]** | **vSideslip [mm/s]** |
| --- | --- | --- | --- | --- | --- |
| 1 | EB imaging,  GCaMP6f6f > P-EN, jRGECO1a > E-PG | 0.65±0.28 | 18±22 | 2.2±1.7 | 1.4±1.6 |
| 2 | “ | 0.44±0.27 | 14±28 | 1.3±1.8 | 0.89±1.6 |
| 3 | “ | 0.58±0.25 | 29±36 | 2.0±1.7 | 1.3±1.7 |
| 4 | „ | 0.63±0.19 | 34±31 | 2.8±1.4 | 1.8±1.7 |
| 5 | “ | 0.56±0.19 | 32±34 | 2.6±1.5 | 1.7±1.7 |
| 6 | “ | 0.37±0.27 | 15±21 | 1.5±1.3 | 0.96±1.3 |
| 7 | „ | 0.56± 0.3 | 28±41 | 1.7±1.8 | 1.1±1.6 |
| 8 | “ | 0.51±0.29 | 20±35 | 1.6±2.2 | 1.0±1.9 |
| 9 | “ | 0.56±0.38 | 25±26 | 3.3±2.4 | 2.1±2.3 |
| 10 | „ | 0.47±0.29 | 26±31 | 2.3±1.9 | 1.5±1.8 |
| 11 | EB imaging,  jRGECO1a > P-EN, GCaMP6f > E-PG | 0.52±0.28 | 14±18 | 1.3±0.92 | 0.78±0.89 |
| 12 | „ | 0.48±0.27 | 15±22 | 1.5± 1.4 | 0.95± 1.4 |
| 13 | „ | 0.31±0.26 | 30±31 | 2.6± 1.6 | 1.6± 1.9 |
| 14 | „ | 0.54±0.29 | 34±37 | 2.9± 1.9 | 1.9± 2.1 |
| 15 | „ | 0.64±0.25 | 27±35 | 2.1±2.0 | 1.3±2.0 |
| 16 | PB imaging,  GCaMP6f6f > P-EN, jRGECO1a > E-PG | 0.52±0.27 | 24±35 | 0.87±1.0 | 0.54±0.95 |
| 17 | „ | 0.53±0.25 | 29±40 | 2.2±2.0 | 1.4±1.9 |
| 18 | „ | 0.52±0.30 | 24±33 | 1.4±1.4 | 0.89±1.4 |
| 19 | „ | 0.50±0.31 | 16±25 | 1.2±1.3 | 0.7±1.3 |
| 20 | „ | 0.39±0.26 | 24±22 | 2.7±2.0 | 1.7±1.8 |
| 21 | PB imaging,  jRGECO1a > P-EN, GCaMP6f > E-PG | 0.72±0.27 | 42±44 | 2.8±1.8 | 1.8±2.0 |
| 22 | „ | 0.70±0.14 | 64±78 | 3.3±2.3 | 2.1±2.4 |
| 23 | „ | 0.55±0.29 | 59±59 | 3.3±1.7 | 2.1±2.1 |
| 24 | „ | 0.54±0.30 | 29±38 | 1.9±1.7 | 1.2±1.7 |
| 25 | „ | 0.41±0.21 | 29±33 | 1.8±1.7 | 1.1±1.8 |
| 26 | „ | 0.42±0.30 | 24±32 | 1.5±1.7 | 0.97±1.6 |
| 27 | PB imaging,  jRGECO1a > P-EN, GCaMP6f > E-PG: VT8135 | 0.42±0.22 | 19±23 | 1.3±1.2 | 0.86±1.3 |
| 28 | „ | 0.45±0.26 | 11±19 | 0.91±1.3 | 0.55±1.1 |
| 29 | „ | 0.49±0.28 | 2.7±12 | 0.15±0.61 | 0.098±0.51 |
| 30 (pre) | Shi^TS^ EB imaging, ShiTS > empty Gal4, GCaMP6f > E-PG: VT25957 | 0.95±0.0097 | 33±36 | 2.7±1.6 | 1.7±1.8 |
| 30 (hot) | " | 1.0±0.0046 | 25±23 | 7.1±2.5 | 4.5±3.0 |
| 30 (post) | " | 0.95±0.079 | 24±27 | 5.0±3.1 | 3.2±2.8 |
| 31 (pre) | " | 0.61±0.052 | 25±36 | 1.6±1.9 | 1.1±1.7 |
| 31 (hot) | " | 0.96±0.033 | 21±20 | 6.4±3.1 | 3.8±3.0 |
| 31 (post) | " | 0.65±0.22 | 15±23 | 1.7±1.9 | 1.1±1.7 |
| 32 (pre) | " | 0.58±0.021 | 18±29 | 1.5±1.9 | 0.94±1.6 |
| 32 (hot) | " | 0.92±0.066 | 21±21 | 6.8±3.7 | 4.2±3.5 |
| 32 (post) | " | 0.95±0.054 | 22±18 | 8.1± 3.3 | 5.2±3.5 |
| 33 (pre) | " | 0.65±0.011 | 16±29 | 1.3±1.6 | 0.85±1.4 |
| 33 (hot) | " | 0.84±0.13 | 19±25 | 1.9±1.5 | 1.2±1.4 |
| 33 (post) | " | 0.69±0.07 | 9.9±15 | 1.2±1.1 | 0.82±1.1 |
| 34 (pre) | " | 0.79±0.11 | 24±32 | 1.8±1.7 | 1.1±1.6 |
| 34 (hot) | " | 0.96±0.061 | 24±22 | 5.3±2.8 | 3.3±2.7 |
| 34 (post) | " | 0.91±0.068 | 17±14 | 3.2±1.9 | 2.1±1.8 |
| 35 (pre) | " | 0.58±0.087 | 21±38 | 1.4±2.1 | 0.91±1.7 |
| 35 (hot) | " | 0.96±0.066 | 23±23 | 5.2±2.8 | 3.3±2.8 |
| 35 (post) | " | 0.54±0.37 | 11±18 | 1.6±2.1 | 1.0±1.6 |
| 36 (pre) | " | 0.62±0.11 | 26±33 | 1.8±1.9 | 1.2±1.8 |
| 36 (hot) | " | 0.82±0.12 | 22±27 | 4.4±3.9 | 2.9±3.3 |
| 36 (post) | " | 0.55±0.10 | 15±26 | 1.2±1.5 | 0.76±1.3 |
| 37 (pre) | " | 0.79±0.034 | 29±33 | 2.3±1.9 | 1.5±2.0 |
| 37 (hot) | " | 0.95±0.033 | 23±22 | 2.8±1.6 | 1.8±1.8 |
| 37 (post) | " | 0.92±0.11 | 18±18 | 3.0±1.6 | 1.9±1.8 |
| 38 (pre) | " | 0.85±0.026 | 41±44 | 2.6±2.1 | 1.6±2.2 |
| 38 (hot) | " | 1.0±0.0039 | 36±28 | 6.1±2.2 | 3.9±2.8 |
| 38 (post) | " | 0.95±0.055 | 32±26 | 5.7±2.6 | 3.7±2.7 |
| 39 (pre) | " | 0.88±0.032 | 30±33 | 2.3±1.7 | 1.5±1.8 |
| 39 (hot) | " | 1.0±0.0090 | 24±22 | 6.9±2.4 | 4.4±3.0 |
| 39 (post) | " | 0.86±0.096 | 25±30 | 3.0±2.3 | 1.9±2.2 |
| 40 (pre) | Shi^TS^ EB imaging,ShiTS > P-EN: VT 8135, GCaMP6f > E-PG: VT25957 | 0.72±0.07 | 21±27 | 1.7±1.7 | 1.1±1.6 |
| 40 (hot) | " | 0.74±0.17 | 17±22 | 2.7±2.5 | 1.7±2.2 |
| 40 (post) | " | 0.14±0.13 | 2.4±9.5 | 0.25±0.82 | 0.17±0.68 |
| 41 (pre) | " | 0.70±0.015 | 21±27 | 1.4±1.5 | 0.91±1.4 |
| 41 (hot) | " | 0.91±0.076 | 25±27 | 3.7±2.4 | 2.4±2.3 |
| 41 (post) | " | 0.81±0.18 | 16±19 | 2.7±2.3 | 1.8±2.0 |
| 42 (pre) | " | 0.86±0.036 | 25±27 | 2.1±1.6 | 1.3±1.6 |
| 42 (hot) | " | 0.99±0.0070 | 27±24 | 5.2±2.5 | 3.3±2.7 |
| 42 (post) | " | 0.17±0.11 | 2.8±9.1 | 0.3±0.87 | 0.18±0.63 |
| 43 (pre) | " | 0.43±0.043 | 12±22 | 0.83±1.3 | 0.55±1.2 |
| 43 (hot) | " | 0.84±0.070 | 17±22 | 2.3±2.1 | 1.4±1.9 |
| 43 (post) | " | 0.13±0.054 | 1.7±8.0 | 0.13±0.51 | 0.095±0.46 |
| 44 (pre) | " | 0.86±0.061 | 16±22 | 1.4±1.3 | 0.91±1.3 |
| 44 (hot) | " | 0.82±0.089 | 10±16 | 1.3±1.3 | 0.86±1.2 |
| 44 (post) | " | 0.1±0.14 | 0.93±5.6 | 0.070±0.33 | 0.048±0.28 |
| 45 (pre) | " | 0.77±0.039 | 16±21 | 1.5±1.3 | 0.95±1.2 |
| 45 (hot) | " | 0.99±0.028 | 24±25 | 6.5±2.8 | 4.0±2.9 |
| 45 (post) | " | 0.84±0.037 | 19±25 | 2.4±1.8 | 1.5±1.6 |
| 46 (pre) | " | 0.69±0.059 | 17±24 | 1.4±1.4 | 0.82±1.3 |
| 46 (hot) | " | 0.93±0.041 | 32±34 | 4.5±3.1 | 2.8±2.9 |
| 46 (post) | " | 0.17±0.17 | 4.0±14 | 0.32±0.90 | 0.2±0.74 |
| 47 (pre) | " | 0.30±0.11 | 5.8±15 | 0.61±1.2 | 0.37±1.0 |
| 47 (hot) | " | 1.0 | 31±24 | 7.1±2.6 | 4.5±3.2 |
| 47 (post) | " | 0.52±0.28 | 11±18 | 2.1±3.1 | 1.3±2.3 |
| 48 (pre) | " | 0.77±0.051 | 14±20 | 1.5±1.5 | 0.98±1.4 |
| 48 (hot) | " | 0.99±0.0069 | 24±24 | 5.9±2.9 | 3.7±2.9 |
| 48 (post) | " | 0.89±0.079 | 19±18 | 4.7±3.2 | 3.0±2.9 |
| 49 (pre) | Shi^TS^ EB imaging,ShiTS > P-EN: R37.F06, GCaMP6f > E-PG: VT25957 | 0.83±0.012 | 30±37 | 2.4±2.0 | 1.5±1.9 |
| 49 (hot) | " | 1.0 | 22±21 | 8.0±2.0 | 4.9±3.2 |
| 49 (post) | " | 0.83±0.052 | 20±23 | 2.0±1.6 | 1.2±1.6 |
| 50 (pre) | " | 0.88±0.03 | 59±54 | 3.7±2.6 | 2.4±2.6 |
| 50 (hot) | " | 0.98±0.018 | 52±37 | 4.9±2.2 | 3.2±2.6 |
| 50 (post) | " | 0.87±0.014 | 35±34 | 2.2±1.7 | 1.4±1.7 |
| 51 (pre) | " | 0.90±0.020 | 34±35 | 2.8±2.0 | 1.8±2.1 |
| 51 (hot) | " | 0.99±0.013 | 36±33 | 5.9±2.6 | 3.5±2.8 |
| 51 (post) | " | 0.64±0.14 | 16±24 | 1.2±1.4 | 0.76±1.3 |
| 52 (pre) | " | 0.77±0.024 | 36±45 | 2.4±2.4 | 1.5±2.3 |
| 52 (hot) | " | 0.93±0.027 | 36±41 | 3.9±2.6 | 2.5±2.6 |
| 52 (post) | " | 0.34±0.18 | 12±28 | 0.79±1.6 | 0.51±1.3 |
| 53 (pre) | " | 0.88±0.0085 | 33±38 | 2.3±1.8 | 1.4±1.8 |
| 53 (hot) | " | 0.97±0.022 | 29±28 | 3.9±2.0 | 2.5±2.2 |
| 53 (post) | " | 0.57±0.076 | 13±23 | 0.95±1.3 | 0.61±1.1 |
| 54 (pre) | " | 0.94±0.028 | 64±53 | ­­4.1±2.4 | 2.6±2.7 |
| 54 (hot) | " | 0.94±0.032 | 33±31 | 3.0±1.7 | 1.9±2.0 |
| 54 (post) | " | 0.58±0.19 | 19±29 | 1.3±1.6 | 0.88±1.5 |
| 55 (pre) | " | 0.84±0.043 | 27±30 | 2.8±2.0 | 1.8±2.1 |
| 55 (hot) | " | 0.99±0.0084 | 27±28 | 4.1±2.2 | 2.6±2.3 |
| 55 (post) | " | 0.90±0.039 | 25±32 | 2.3±2.1 | 1.4±1.9 |
| 56 (pre) | " | 0.85±0.030 | 27±33 | 2.1±1.8 | 1.3±1.8 |
| 56 (hot) | " | 0.99±0.0096 | 33±34 | 4.8±2.3 | 3.1±2.5 |
| 56 (post) | " | 0.68±0.12 | 20±29 | 1.4±1.6 | 0.91±1.4 |
| 57 (pre) | " | 0.88±0.082 | 43±46 | 3.2±2.1 | 2.0±2.3 |
| 57 (hot) | " | 0.83±0.075 | 22±28 | 2.5±1.9 | 1.6±1.8 |
| 57 (post) | " | 0.78±0.075 | 12±15 | 1.7±1.3 | 1.1±1.2 |
| 58 (pre) | " | 0.89±0.085 | 40±41 | 2.5±2.0 | 1.6±2.0 |
| 58 (hot) | " | 0.66±0.10 | 18±29 | 1.3±1.6 | 0.82±1.4 |
| 58 (post) | " | 0.40± 0.14 | 6.3±13 | 0.45±0.78 | 0.29±0.69 |
